# Transcutaneous electrical acupoint stimulation for high-normal blood pressure: study protocol for a randomized controlled pilot trial

**DOI:** 10.1186/s13063-021-05039-5

**Published:** 2021-02-15

**Authors:** Yu Wang, Guang-Xia Shi, Zhong-Xue Tian, Jun-Hong Liu, You-Sheng Qi, Jian-Feng Tu, Jing-Wen Yang, Li-Qiong Wang, Cun-Zhi Liu

**Affiliations:** 1 International Acupuncture and Moxibustion Innovation Institute, School of Acupuncture-Moxibustion and Tuina, Beijing University of Chinese Medicine, Chaoyang District, No. 11, Bei San Huan Dong Lu, Chaoyang District, Beijing, 100029 China; 2 Nanyuan Community Health Service Center, Fengtai District, Beijing, China; 3 Department of Acupuncture and Moxibustion, Beijing Hospital of Traditional Chinese Medicine Affiliated to Capital Medical University, Dongcheng District, Beijing, China

**Keywords:** Transcutaneous electrical acupoint stimulation, High-normal blood pressure, Lifestyle interventions, Prehypertension

## Abstract

**Background:**

High-normal blood pressure (BP) is associated with increased all-cause, cardiovascular mortality and frequently progresses to hypertension. Transcutaneous electrical acupoint stimulation (TEAS) might be a non-pharmaceutical therapy option to control BP. This trial aims to determine the effectiveness and safety of TEAS combined with lifestyle modification for high-normal BP.

**Methods/design:**

This prospective, randomized, and parallel clinical trial will be conducted in a community service center in China. Sixty participants with high-normal BP will be randomly allocated to receive TEAS plus lifestyle modification (intervention group) or lifestyle modification alone (control group) in a 1:1 ratio. In addition to lifestyle modification, the intervention group will receive TEAS at four acupoints for 30 min, 4 times weekly for 12 weeks for a total of 48 sessions at home. The control group will receive same lifestyle modification but no TEAS. The primary outcome will be the change in mean systolic blood pressure at 12 weeks from the baseline measurement. Secondary outcomes include the change of mean diastolic blood pressure, proportion of subjects with progression to hypertension, quality of life, body mass index, and waist circumference. Adverse events during the trial will be monitored.

**Discussion:**

This trial will explore the feasibility and provide potential evidence for the effectiveness and safety of TEAS plus lifestyle modification for high-normal BP. Furthermore, this pilot trial is being undertaken to determine the feasibility of a full scale definitive randomized controlled trial. The results of this study will be published in a peer-reviewed journal.

**Trial registration:**

Chinese Clinical Trial Registry, ChiCTR 1900024982. Registered on August 6, 2019.

**Supplementary Information:**

The online version contains supplementary material available at 10.1186/s13063-021-05039-5.

## Background

Increased blood pressure (BP) is a leading modifiable risk factor for global disease burden and premature mortality [1, 2]; even mild BP elevations manifesting as “prehypertension or high-normal BP” have been associated with increased all-cause mortality [3]. High-normal BP elevates the risk of higher morbidity and mortality from cardiovascular disease (CVD) and stroke [4, 5], as well as increasing the risk of incident end-stage renal disease [6]. High-normal BP frequently progresses to hypertension [7], and affects approximately 41.3% (estimated 435.3 million) of adults in China according to the Chinese guideline [8]. High-normal BP may be a window of opportunity to prevent hypertension and its cardiovascular consequences [9]. The 2018 Chinese Guidelines for Prevention and Treatment of Hypertension defined high-normal BP as systolic blood pressure (SDP) of 120–139 mmHg and/or diastolic blood pressure (DBP) of 80–89 mmHg [10]. However, the 2017 American College of Cardiology (ACC)/American Heart Association (AHA) BP guideline defined stage 1 hypertension as SBP of 130–139 mmHg or DBP of 80–89 mmHg [11]. Therefore, a large number of individuals who had prehypertension or high-normal BP before are now considered to have hypertension according to the new American guideline.

Data are lacking as to how this range of BP should be managed and most guidelines do not recommend pharmacological interventions for high-normal BP without cardiovascular comorbidity [7]. Long-term medication is costly to control BP particularly in developing nations, for instance, the price in China was 3.3 times the price in the USA on average [12]. Despite limited effectiveness, virtually, all guideline statements recommend and encourage lifestyle interventions to prevent hypertension [7]. However, lifestyle modification is a dynamic process and requires long-term persistence [13], and the challenges of lifestyle changes extend beyond counseling for physicians and patient adherence [14]. For the majority of US adults with a SBP of 130–139 or DBP of 80–89 mmHg, non-pharmaceutical therapy by itself is the recommended treatment [15].

Acupuncture as a non-pharmaceutical therapy is widely used in the treatment of hypertension [16], and some evidence suggests potential effectiveness [17]. Transcutaneous electric acupoint stimulation (TEAS), a noninvasiveness therapy similar to acupuncture, is used as a clinical alternative to electrical acupuncture and manual acupuncture [18]. It is considered a combination of transcutaneous electrical nerve stimulation and acupuncture. Transcutaneous electrical stimulation therapy has the advantages of being easy to use, which is more “user friendly” requiring minimal training for physicians and patients, and if convenient for clinical application [19]. Two trials have explored the efficacy of simple transcutaneous electrical nerve stimulation based interventions for adults with or without hypertension to reduce blood pressure [20, 21]. And choosing acupoints with antihypertensive effect based on traditional Chinese medicine theory for stimulation may increase the effect of transcutaneous electrical nerve stimulation. Previous studies have shown that TEAS has effects on the nervous system that include regulation autonomic nervous system function and enhancement of the activity of the vagus nerve, thereby potentially affecting BP [22]. This approach was supported by the studies of Jacobsson et al. [23] and Zhang et al. [24] using the Hans electrical stimulator treating acupoints on the forearm showed significant reduction in blood pressure. However, studies of TEAS for high-normal BP are lacking. The main objective of this preliminary trial is to assess the effectiveness and safety of TEAS combined with lifestyle modification in participants with high-normal BP, to calculate the sample size for a future efficacy study.

## Methods

### Study design

This prospective, randomized, and parallel design clinical trial of high normal BP participants will be conducted in a large public-sector clinic in Beijing, China. The trial protocol was approved and reviewed by the Ethics Committee of Beijing University of Chinese Medicine and will be reported based on SPIRIT guidelines. The corresponding author will be responsible for trial scientific oversight. The study was registered on Chinese Clinical Trial Registry (ChiCTR 1,900,024,982) on August 6, 2019.

### Study population

The study population will be comprised of individuals with high-normal BP (2018 Chinese Guidelines for Prevention and Treatment of Hypertension [10]) at a community service center (Nanyuan community health service centers in Beijing, China). Study investigators will be responsible for recruiting and gaining informed consent. All participants will provide written informed consent before enrollment and randomization at the outpatient department.

#### Inclusion criteria

1. Aged between 35 and 65 years (either gender)
2. SBP of 120–140 mmHg and/or DBP of 80–90 mmHg on at least 2 separate visits
3. No language disorder or mental retardation, so that participants will be able to answer and complete the questionnaire completely
4. Willing to sign written informed consent

#### Exclusion criteria

1. Contraindications for the use of electrostimulation such as use of a cardiac pacemaker or other implanted medical devices; suffering from acute diseases, infectious diseases, malignant tumors, cardiovascular disease, cerebrovascular disease, liver and kidney dysfunction, or other malignant diseases; dermatological abnormalities on the skin of the acupuncture points
2. Secondary hypertension
3. Received antihypertensive drugs or other drugs that affect BP in the previous 2 months
4. Uncontrolled diabetes
5. Received acupuncture treatment in the previous 1 month
6. Drug or alcohol abuse
7. Pregnant, lactating, or planning pregnancy during the trial
8. Participated in another research trial

### Randomization and allocation concealment

Eligible participants will be randomized into one of two groups: the intervention group or the control group (1:1), using block random method and the block size is 6. The randomization sequence will be prepared by a professional statistician (Na Zhang) with the SAS9.3 software, who is not involved in assessment, treatment, or analysis of the study, to ensure balance in baseline BP across the groups. When an eligible participant needed to receive a random group, the random number and the group assignment will be sent from the random number administrator to the recruiter via phone or short message.

The participants and study staff interacting with participants will not be blinded to group assignment in the trial. The outcome assessors and trial statisticians, who will not be involved in the intervention, will be blinded.

### Interventions

Transcutaneous electrical stimulation will be applied to the acupuncture points in the intervention group, and the control group will not have TEAS. Based on the nature of chronic disease and barrier of time necessary for frequent transportation to hospital, the majority of treatment during 12 weeks will be operated by participants themselves at home. And participants may distinguish whether the instrument is on or not. It is difficult to blind the participants. Hence, we do not set sham therapy as the control group. Both groups will be educated about lifestyle modification because the guidelines clearly recommend that lifestyle changes should be basic treatments at high-normal blood pressure.

#### TEAS

A household transcutaneous nerve stimulator (SDP-330; Yuwell, Suzhou Medical Appliances Co, Ltd., Suzhou, China) with two 100-Hz output channels at a pulse width of 0–100 μs will be used for the application of TEAS. It has 10 different stimulus intensities, and the intensity of the stimulation will be individually adjusted by the participants with the recommendation to increase the intensity gradually to trigger the maximum sensory threshold without discomfort or pain. The self-adhesive electrodes measuring 5 × 5 cm will be placed on the acupuncture points region. The stimulator has 8 different modes, but the participants will be asked to select one of the “press” or “knock” or “knead” mode, which cannot be changed during treatment. The participants will be asked to keep a treatment diary and register the time of stimulation.

**Fig. 1:**
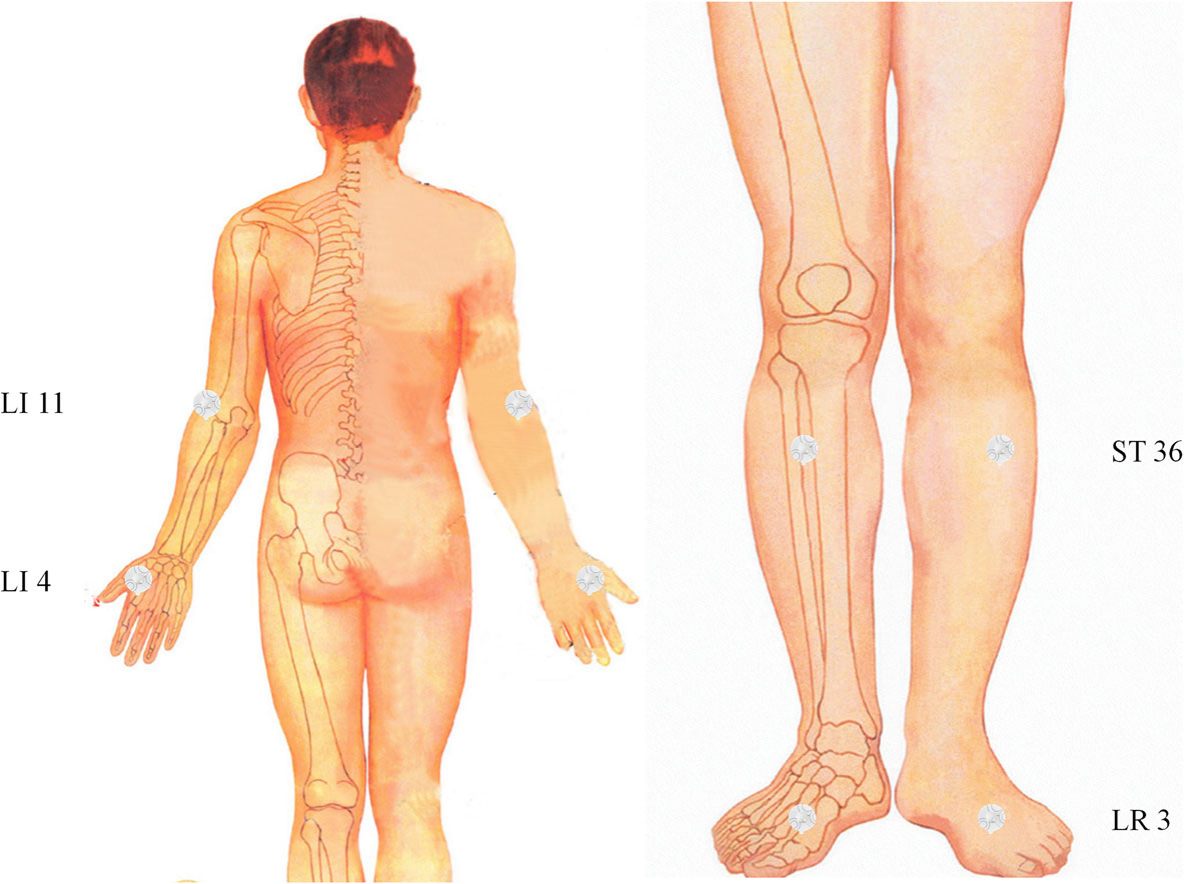
Four bilaterally acupoints with relevance for BP lowering according to traditional Chinese medicine concepts

The following four bilaterally acupoints with relevance for BP lowering according to traditional Chinese medicine concepts determined by a literature review [25] will be used in the intervention group: *Hegu* (LI4), *Quchi* (LI11), *Zusanli* (ST36), and *Taichong* (LR3) (Table 1 and Fig. 1). Traditional Chinese medicine believes that *Yin* and *Yang* rise and fall orderly, harmonize of *Qi* and blood, and normalize blood pressure under normal physiological conditions. Worry and anger, improper diet, or fatigue and internal injury can cause the imbalance of liver and kidney, *Yin* and *Yang*, viscera dysfunction, endogenous wind and heat, and *Qi* and blood adversity and raise blood pressure. The above acupuncture points are considered to regulate viscera, balance *Yin* and *Yang*, harmonize *Qi* and blood, and regulate blood pressure to restore normal [26]. Some of the acupoints were supported by previous studies which used the TEAS treating *Hegu* and *Quchi* on the forearm and showed significant reduction in blood pressure [23, 24]. In previous study, we founded that acupuncture at *Taichong* decreases high blood pressure and nicotinamide adenine dinucleotide phosphate oxidase in the rostral ventrolateral medulla of spontaneously hypertensive rats [27], and the other study suggested that the beneficial effects of electroacupuncture at *Zusanli* on hypertension may be through modulation of functional γ-aminobutyric acid receptors in the nucleus tractus solitarii [28]. The clinical efficacy of these two acupoints has also been confirmed in a multicenter clinical trial [29]. Furthermore, these 4 points were on the limbs so it is very easy for participants to access and apply treatments.

**Table 1:** Locations of acupoints in intervention group

Acupoints	Locations
*Hegu* (LI4)	On the dorsum of the hand, radial to the midpoint of the second metacarpal bone.
*Quchi* (LI11)	On the lateral aspect of the elbow, at the midpoint of the line connecting LU5 with the lateral epicondyle of the humerus.
*Zusanli* (ST36)	3 cun^a^ directly below *Dubi* (ST35), and one finger-breadth lateral to the anterior border of the tibia.
*Taichong* (LR3)	In the depression anterior to the junction of first and second metatarsal bones.

^a^1 cun (≈20 mm) is defined as the width of the interphalangeal joint of patient’s thumb

The first treatment will be at LI4 and LI11 on the same arm for 15 min, followed by the same acupoints on the opposite arm for 15 min. The second treatment will be at ST36 and LR3 on the same leg for 15 min, and then the same acupoints on the opposite leg will be taken for 15 min. Each treatment will last a total of 30 min, and different acupoints will be used. Participants will be asked to alternate treatment on alternate days to guarantee they will receive 4 times TEAS per week and 48 times in total and cannot increase or decrease the number of times.

**Fig. 2:**
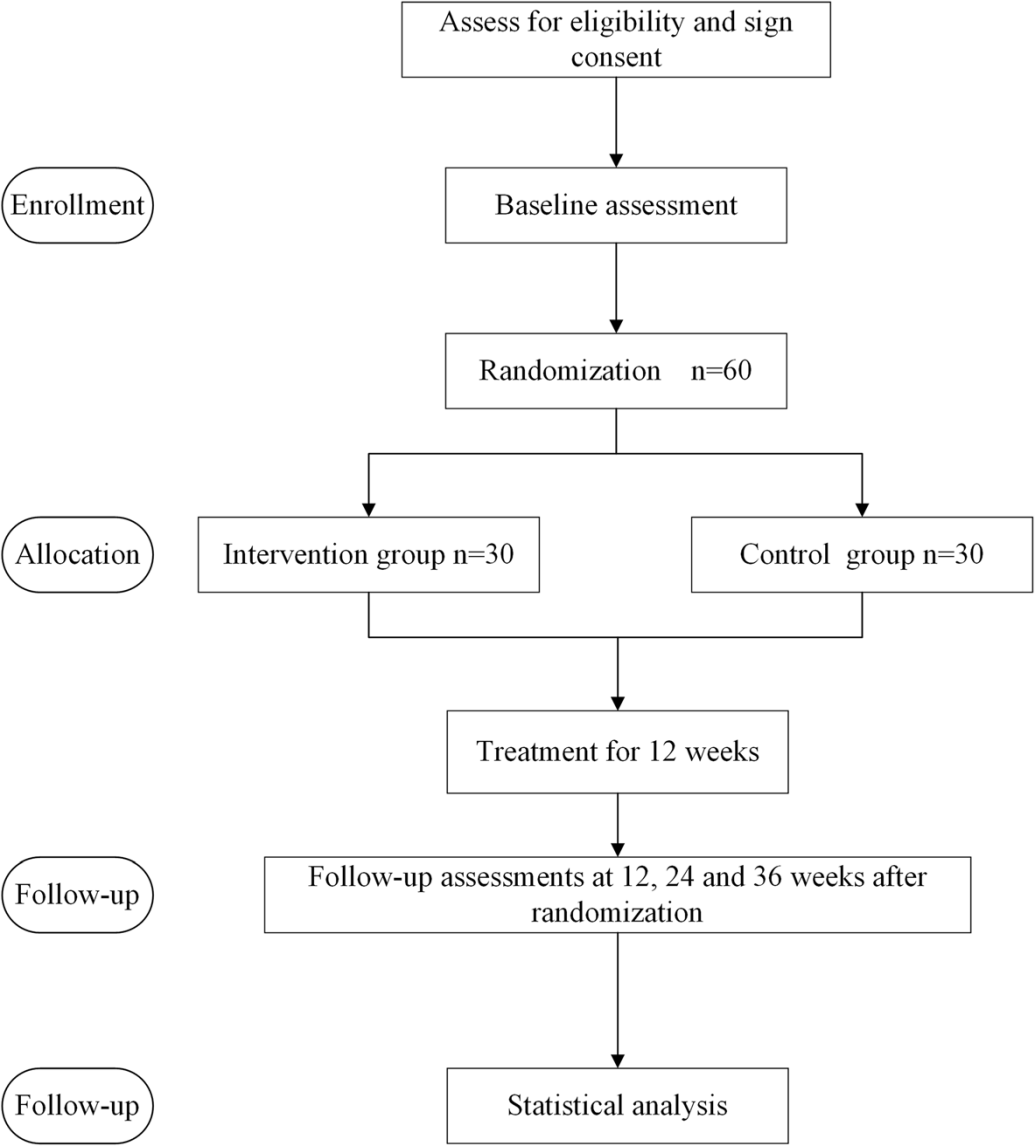
Study design

Each participant in the intervention group will receive a stimulator with written instructions as well as the participants’ manual on how to do the TEAS treatment properly. Treatment will be performed at the participants’ home, and TEAS will be performed by the participants. At the beginning of the trial, the study investigators will instruct the participants to locate the acupoints. Participants will be asked to take photos to provide feedback to investigators during the initial treatment, to ensure that participants can accurately find all 4 acupoints. In addition, acupoint pictures and videos will be produced to assist participants. Study investigators will ensure the participants fully understand the operation of TEAS and the location of acupoints. Figure 2 shows the study design.

#### Lifestyle modification

Participants in both groups will receive recommendation for lifestyle modification. All participants will receive relevant weekly information through the WeChat app (Tencent, Shenzhen, China) in mobile phone and monthly educational activities in the community. They contain information about weight control, increase physical activity, healthy eating, dietary sodium reduction, smoke abatement, and set limit to alcohol. This approach was supported by previous study which used a WeChat-based lifestyle modification in China and showed significant reduction in blood pressure for patients with hypertension [30].

### Outcomes

#### Primary outcome

The primary outcome will be the change in mean SBP from baseline to 12 weeks.

BP will be measured as proposed by the 2018 Chinese Guidelines for Prevention and Treatment of Hypertension [10]. Participants will be asked to avoid exercise, alcohol, cigarettes, and coffee/tea for at least 30 min before the BP measurement. BP will be measured with the participant in a seated position after 5 min of quiet rest. Participants will be required to take blood pressure measurement. A digital BP monitor (HEM-7136, OMRON Corporation, Kyoto, Japan) with suitable cuff size will be used. Clinicians will record three sequential BP readings at 5-min intervals, and the final BP will be calculated by removing the initial reading and calculating the mean from the two remaining readings.

#### Secondary outcomes

##### Changes in mean blood pressure at other time points

Other secondary outcomes include measures of changes in mean SDP and DBP from baseline to 4, 8, 12, 24, and 36 weeks.

##### Proportion of progression to hypertension

The proportion of subjects with hypertension (BP > 140/90 mmHg) will be calculated among the high-normal BP participants at 12, 24, and 36 weeks.

##### Quality of life (QoL)

The 12-item Short Form Health Survey (SF-12) [31] will be used for quality of life at baseline, 12, 24, and 36 weeks. The questionnaire consists of a mental domain and a physical domain (each domain ranges from 0 to 100), and a higher score will be considered to indicate a better quality of life.

##### Body mass index (BMI) and waist circumference

Net change in BMI and waist circumference will be measured at baseline, 12, 24, and 36 weeks. The BMI, defined as the weight (kg) divided by the participant’s height squared (m^2^) [32], will be calculated as an index for obesity. And the waist circumference will be measured (at the smallest circumference between the iliac crest and the lower costal margin) in centimeters (cm).

##### Adverse events (AEs)

**Fig. 3:**
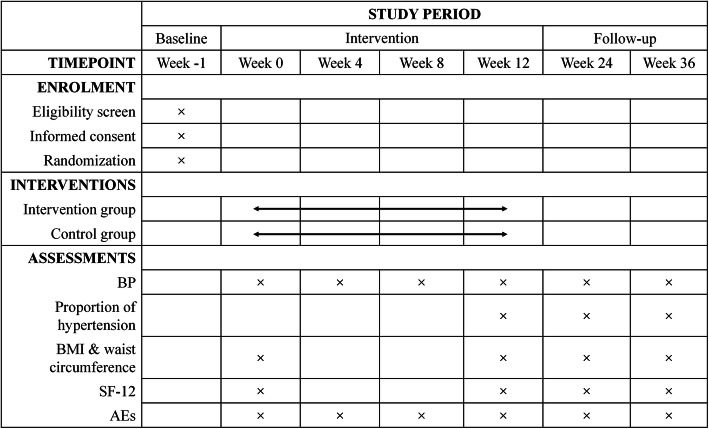
Schedule of enrolment, intervention and assessments

Any AEs will be monitored and documented throughout the trial by the investigators and participants. Based on their potential association with the TEAS procedure, AEs will be categorized by specialists as treatment-related or non-treatment-related within 24 h of occurrence. Potential AEs of TEAS used in the trial include continuous post-electrostimulation sensation and skin numbness. The schedule of enrolment, intervention, and assessments is shown in Fig. 3. If acute or uncontrolled hypertension or other serious adverse reactions occur, the participants will be asked to discontinue from this trial and seek medical attention immediately.

##### Quality assurance and quality control

To guarantee the quality of the study, the trial protocol was reviewed and revised by experts in hypertension, acupuncture, methodology, and statistics. A pre-specified standard operating procedure of BP measurement, including selection of appropriate cuffs, reading and calculation of mean BP, and maintaining and calibrating of electronic sphygmomanometer, will be developed to reduce measurement error. Other standardized procedures for the operational aspects of the study including details in filling out questionnaires, recruitment, lifestyle intervention coaching, assessment of AEs, and data management will also be used to train the study personnel. The clinical research associate (CRA) will review the data regularly, for authenticity and timeliness of data collection and data quality. All data will be collected onto paper questionnaires and then transferred to an Excel spreadsheet and will be preserved for at least 5 years after publication of the trial results. Hence, all data collected during the course of the research will be kept strictly confidential and only accessed by the study staff. The private information of patients including the name and telephone number will be critically protected and will never be allowed to be disclosed. If reviewers or readers have any questions regarding our published data, or if any researcher will conduct meta-analyses, they can contact the corresponding author for access to the anonymized data. Detailed instructions, acupoint pictures, videos, and treatment procedures of the TEAS will be produced and distributed to each participant in the intervention group. Study investigators will ensure participants’ compliance with the intervention protocol. Participants will be registered with a phone number and address for further contact in case of missing outlined visits.

### Statistical methods

#### Sample size

This pilot study aims to assess the effectiveness and safety of TEAS combined with lifestyle modification for high-normal BP and determine the feasibility of a further large clinical trial. The minimum sample size for exploratory trials is 20 to 30 per group according to Provisions for Drug Registration in China. It is generally accepted that at least 30 participants are required for a pilot study [33]. We selected the maximum of 30 participants, and the sample size of 60 participants was determined. The results of this study will facilitate the calculation of an appropriate sample size for further randomized clinical trials.

#### Statistical analysis

Analysis will be carried out on an intention-to-treat (ITT) basis and will be performed using SPSS 23.0 statistical software (IBM SPSS Statistics, New York, USA) with a 2-sided *P* value of less than 0.05 considered significant. The measurement of data that conforms to the normal distribution will be expressed as mean ± SD, the measurement data that does not conform to a normal distribution will be expressed by the median (interquartile range), and counting data will be represented by cases (percentages). The continuous variables will be evaluated by using a *t* test or the Mann-Whitney *U* test for comparison. The chi-square test or Fisher’s exact test will be employed to compare binary variables. Missing data will be imputed using the last observation carried forward.

## Discussion

This manuscript presents the design of a randomized controlled pilot trial testing the effectiveness and safety of TEAS combined with lifestyle modification to improve BP control. To the best of our knowledge, there is no literature that has reported the effectiveness of TEAS combined with lifestyle modification on high-normal BP has been published.

Numerous studies have confirmed that high-normal BP is highly prevalent and increases the risk of incident hypertension, cardiovascular events, and death [7]. In China and other low-income and middle-income countries, long-term medication is costly to control BP. The intervention for the trial is innovative. For individuals with high-normal BP, exploring non-pharmaceutical therapies is feasible and necessary. Domestic TEAS may be an appropriate form of intervention. TEAS will be performed by the participants themselves at home, reducing transportation costs and possibly increasing compliance. Study investigators will ensure the participants fully understand the operation of TEAS. The education of lifestyle modification based on a commonly used social media software is also low cost. Therefore, the intervention model is characterized by accessibility, flexible, and low cost.

A limitation in the present study is the failure to clarify the optimal frequency of electrical stimulation of TEAS. Second, the participants are not blinded to the nature of intervention. At the end of this pilot trial, the results will potentially provide evidence of the feasibility and effectiveness of TEAS combined with lifestyle modification for high-normal BP and, if effective, a future large clinical definitive randomized controlled trial will be conducted.

### Trial status

Protocol: version 2.0, 30 June 2019.

The first patient was recruited on 6 September 2019. At the present time, a total of 60 patients had been randomized. The final date of follow-up is expected to be November 2020. This protocol was submitted prior to the recruitment of total 60 patients.

## Supplementary Information

**Additional file 1.** Completed Standard Protocol Items: Recommendation for Interventional Trials (SPIRIT) 2013 Checklist: items addressed in this clinical trial protocol.

## Data Availability

The datasets analyzed during the current study are available from the corresponding author on reasonable request.

## Abbreviations

TEAS: Transcutaneous electrical acupoint stimulation
BP: Blood pressure
CVD: Cardiovascular disease
SDP: Systolic blood pressure
DBP: Diastolic blood pressure
SF-12: 12-Item Short Form Health Survey
IPAQ: International Physical Activity Questionnaire
BMI: Body mass index
AEs: Adverse events
CRA: Clinical research associate
ITT: Intention-to-treat
